# Deciphering the immune landscape dominated by cancer-associated fibroblasts to investigate their potential in indicating prognosis and guiding therapeutic regimens in high grade serous ovarian carcinoma

**DOI:** 10.3389/fimmu.2022.940801

**Published:** 2022-09-02

**Authors:** Yimin Li, Ruotong Tian, Jiaxin Liu, Juanni Li, Hong Tan, Qihui Wu, Xiaodan Fu

**Affiliations:** ^1^ Department of Pathology, Xiangya Hospital, Central South University, Changsha, China; ^2^ Department of Pathology, Fudan University Shanghai Cancer Center, Shanghai, China; ^3^ Department of Oncology, Shanghai Medical College, Fudan University, Shanghai, China; ^4^ Department of Pharmacology, School of Basic Medical Sciences, Shanghai Medical College, Fudan University, Shanghai, China; ^5^ Department of Pathology, School of Basic Medical Sciences, Central South University, Changsha, China; ^6^ National Clinical Research Center for Geriatric Disorders, Xiangya Hospital, Changsha, China; ^7^ Department of Pathology, The Second Xiangya Hospital, Central South University, Changsha, China; ^8^ Department of Obstetrics and Gynecology, Xiangya Hospital, Central South University, Changsha, China

**Keywords:** high grade serous ovarian carcinoma, cancer-associated fibroblast, tumor immune microenvironment, prognosis, therapy prediction

## Abstract

Limited immunotherapeutic effect in high-grade serous ovarian carcinoma (HGSOC) propels exploration of the mechanics behind this resistance, which may be partly elucidated by investigating characters of cancer-associated fibroblasts (CAFs), a significant population in HGSOC involved in shaping tumor immune microenvironment. Herein, leveraging gene expression data of HGSOC samples from The Cancer Genome Atlas and Gene Expression Omnibus datasets, we suggested that CAFs detrimentally affected the outcomes of HGSOC patients. Subsequently, we performed weighted gene co-expression network analysis (WGCNA) to identify a CAFs-related module and screened out seven hub genes from this module, all of which were positively correlated with the infiltration of immunosuppressive macrophages. As one of the hub genes, the expression of fibrillin 1 (FBN1) and its relevance to CD206 were further verified by immunohistochemistry staining in HGSOC samples. Meanwhile, we extracted genes that correlated well with CAF signatures to construct a CAFscore. The capacity of the CAFscore as an independent prognostic factor was validated by Cox regression analyses, and its relevance to components as well as signals in the tumor immune microenvironment was also investigated. Under the evaluation by the CAFscore, HGSOC patients with relatively high CAFscore had worse outcomes, activated mesenchymal signaling pathways, and immune checkpoint blockade (ICB) resistance signatures, which was consistent with the fact that non-responders in anti-PD-1 treatment cohorts tended to have higher CAFscore. Besides, the possibility of CAFscore to guide the selection of sensitive chemotherapeutic agents was explored. In conclusion, individualized assessment of the CAFscore could uncover the extent of stroma activation and immunosuppression and inform therapeutic strategies to improve the benefit of therapies.

## Highlights

From the view of the intricate interplay between CAFs and the immune microenvironment of HGSOC, we identify a gene module associated with CAF traits and generate a CAFscore evaluation system. As an independent prognostic factor, the CAFscore extensively contacts with components and signals in the HGSOC microenvironment. Evaluating the CAFscore of individual may contribute to gaining a greater understanding of stroma and the immune status of each patient, enhancing the accuracy of prognostic prediction, and suggesting effective treatment options.

## Introduction

Ovarian carcinoma is the most fatal of all gynecologic cancers, of which high-grade serous ovarian carcinoma is characterized by a high recurrence rate with poor long-term survival and results in the highest death tolls (1–3). Its malignant biological properties are reflected in its early and widespread dissemination to peritoneal surfaces, which largely relies on communication between tumor cells and their adjacent stromal microenvironment. Previous studies including The Cancer Genome Atlas (TCGA) have identified various subtypes of HGSOC, among which the mesenchymal subtype was linked to conspicuously poorer survival when compared with other subtypes, with increased stromal components such as myofibroblasts and microvascular pericytes (4, 5), highlighting the importance of the tumor stroma for the survival of HGSOC patients.

Cancer-associated fibroblasts (CAFs), originating from diverse groups of mesenchymal cells, are a prominent stromal population in almost all tumors (6). Multiple mechanisms, such as inflammatory signals, DNA damage, and physiological stress, can lead to CAFs activation (7). Through secreting growth factors, inflammatory ligands, and extracellular matrix (ECM) proteins, activated CAFs extensively interact with cancer cells and exert protumorigenic and antitumorigenic effects. In the past decade, the adverse effects of CAFs on ovarian cancer have mostly been illustrated. Functionally, ovarian tumor cells activate fibroblasts or induce cancer-associated fibroblasts-phenotype to promote ovarian cancer progression and reduce overall survival by secreting lysophosphatidic acid, interleukin-1β (IL-1β), and C-C motif chemokine ligand 5 (CCL5) (8–10). Reciprocally, activated ovarian CAFs contribute to epithelial ovarian cancer metastasis by promoting angiogenesis and tumor cell invasion, and even the resistance to platinum-based chemotherapy, through releasing growth factors and metabolites (11–13). Prior studies have shown a predominance of the fibroblast in the HGSOC patient samples, based on the analysis of single-cell separation and sequence (14, 15). Remarkably, myofibroblasts and cancer-associated fibroblasts driven by transforming growth factor-β (TGF-β) predicted the poor outcome of HGSOC patients (16). As such a major component of the stroma, CAFs affect each cancer developmental stage, from initiation to invasion and metastasis, leading to an unfavorable prognosis of ovarian cancer, which inspired us to develop a prognostic model of ovarian cancer based on the status and content of CAFs.

In addition to considering the interactions between CAFs and tumor cells, CAFs’ engagement in crosstalk with other cells within the tumor microenvironment (TME) also deserves attention. The contribution of CAFs to establishing an immunosuppressive TME has been supported by several lines of evidence. In detail, CAFs not only impaired the functionality of dendritic cells (DCs) and the infiltration of natural killer (NK) cells but also facilitated the immunoinhibitory phenotype of macrophages and the differentiation of naïve T cells into regulator T cells (Tregs) (17–20). The potential intervention of fibroblasts in TME is not limited to expressing ligands of immune checkpoint molecules (ICMs), including programmed death ligand 1 (PD-L1), PD-L2, and B7-H3/H4 on their own surface (21). Also, CAFs upregulates the expression of ICMs on other cells in the TME, thereby contributing to the impaired function of tumor-infiltrating T lymphocytes. In the past decade, immune checkpoint blockade, which has revolutionized the treatment of several cancer types shows only modest results in HGSOC (22–25). Nevertheless, little is known about the molecular mechanisms that dictate response or resistance to these modalities. Considering the above backgrounds, investigating interactions between CAFs and the immune microenvironment helps elucidate the mechanism beneath the limited effectiveness of immunotherapies in HGSOC and develop CAFs-targeting immunotherapies.

Herein, prognosis-oriented clustering analysis distinguishing two groups of HGSOC patients with a significant difference in CAFs infiltration suggested that CAFs were significantly involved in the outcomes of HGSOC. Leveraging global gene expression data from several independent sets of clinical HGSOC tumor samples, we identified a gene co-expression module that presents high correlations with signatures of CAFs and significantly overlaps with participators of ECM. Subsequently, we screened out seven hub genes from this CAF-related module, among which FBN1 was further verified by immunohistochemistry (IHC) staining of HGSOC samples. To a large extent, these hub genes might be interpreted as fibroblast markers and correlated well with macrophage infiltration. Meanwhile, we extracted genes that correlated well with CAF signatures to construct a CAFscore. Under the evaluation by the CAFscore, HGSOC patients with relatively high CAFscore had worse outcomes, activated mesenchymal signaling pathways, and ICB resistance signatures, which was consistent with the fact that non-responders in anti-PD-1 treatment cohorts tended to have higher CAFscore. Besides, the possibility of CAFscore to guide chemotherapeutic drug selection was explored.

## Materials and methods

### Dataset acquisition and preprocessing

The R package “TCGAbiolinks” was used to download TCGA RNA-seq data (FPKM normalized), and clinical data were obtained from the cBioPortal website (http://www.cbioportal.org/). Then, the FPKM values were transformed into transcripts per kilobase million (TPM) values. The RNA sequencing data and clinicopathological characteristics of TCGA pan-cancer were obtained from UCSC Xena (https://xenabrowser.net/datapage/). For the HGSOC cohort, the expression data and detailed clinical information of GSE140082, GSE17260, GSE18520, GSE26193, GSE30161, and GSE32063 were downloaded from the Gene Expression Omnibus (GEO) (http://www.ncbi.nlm.nih.gov/geo/). Our study included two immune checkpoint blockade treatment cohorts with available expression and clinical information: the IMvigor210 cohort (obtained from http://research-pub.Gene.com/imvigor210corebiologies) and the GSE78220 cohort (downloaded from GEO). The data preprocessing methods were previously reported (26). Only patients with complete related information were included in each cohort above in this study. Batch effects from non-biological technical biases were corrected using the “ComBat” algorithm of the “sva” package.

To further verify the expression of relevant key genes, 41 HGSOC samples were collected from Xiangya Hospital of Central South University and written informed consent was obtained from the Xiangya Hospital Ethics Committee. The patients were informed and signed informed consent forms.

### Estimation of TME cell infiltration

The immune score, stromal score, ESTIMATE score, and tumor purity for tumor samples were estimated using the R package “ESTIMATE” (27). Meanwhile, the levels of infiltrating CAFs that was calculated by EPIC, MCPcounter and tumor immune dysfunction and exclusion (TIDE), and other immune cells that were calculated by CIBERSORT, EPIC, TIMER, and MCPcounter algorithms in the TME of ovarian cancer (27–31).

### Weighted gene co-expression network analysis

In this study, we conducted weighted gene co-expression analysis (WGCNA) using the R package “WGCNA” to cluster gene modules most correlated with CAFs based on EPIC, MCPcounter, and TIDE (32). We selected a soft threshold power β=3 and then constructed the adjacency matrix by raising the intergenic Pearson correlation matrix to the soft threshold power. The correlation between each module and the different CAFs groups was further selected by selecting the modules with the highest module-CAFs associations to further select candidate modules related to CAFs infiltration. Module membership (MM) represented the correlation between module eigengenes and gene expression profiles, while gene significance (GS) was defined as the absolute value of the correlation between the gene and the clinical trait.

### Generation of the CAFscore

The WGCNA was used to recognize co-expressed gene modules closely related to the CAFs, and a total of 145 genes were determined in the brown gene module with GS>0.3 and MM>0.6. Then, we used the ssGSEA (Single-sample Gene Set Enrichment Analysis) algorithm to construct a CAF-relevant gene signature to quantify the content of the CAFs of individual patients.

### Functional and pathway enrichment analysis

Gene ontology (GO) and Kyoto Encyclopedia of Genes and Genomes (KEGG) pathway analyses *via* the R package “clusterprofiler” with a strict cutoff value of false discovery rate (FDR)<0.05 (33). We performed gene set variation analysis (GSVA) enrichment analysis as in our previous study (34). The R package “IOBR” constructed a gene set that stored genes associated with some biological processes (35). The stroma pathways, DNA damage repair pathways, and immune-related pathways were downloaded, and the ssGSEA method was chosen in the process of pathway score evaluation (36).

### Immunohistochemistry (IHC) staining

IHC was performed as described previously (37). Primary antibodies against CK (Ready to use, Maxim, MAB-0828), CD206 (1:10000, Proteintech, Cat No.60143-1-Ig), FBN1 (1:500, Proteintech,Cat No.26935-1-AP) were used for IHC staining.

### Association analysis of the CAFscore and Immuno-/Chemotherapeutic Response prediction

We investigated the predictive capacity of CAFscore in responding to immunotherapy and chemotherapies/targeted therapies. First, the TIDE algorithm and the Immune Cell Abundance Identifier (ImmuCellAI) algorithm were used to predict the response to ICB therapy as previously described (38). The drugs’ 50% inhibiting concentration (IC_50_) value was predicted using the “pRRophetic” algorithm, and the correlation between CAFscore and the IC_50_ value of the drugs was determined using Spearman correlation analysis.

### Statistical analysis

The statistical difference in the distribution in the two groups was examined by unpaired Student’s t-tests (normally distributed) and the Wilcoxon rank-sum test (nonnormally distributed). Pearson’s or Spearman’s correlation analysis was used to examine the relationships between two continuous variables. The chi-square and Fisher’s exact tests were adopted to analyze the difference between categorical variables. We used the R package “Survminer” to determine the optimal cutoffs, and samples were classified into high and low score groups based on the cutoff. Then, survival analysis was carried out using the Kaplan-Meier method, and the log-rank test was utilized to calculate the statistical significance. A univariate Cox regression model was adopted to calculate the hazard ratios (HR) for CAFs, and a multivariable Cox regression model was used to ascertain the independent prognostic factors. All statistical analyses were conducted using R software (version 4.0.5). The *p* values were two-sided, and *p* values < 0.05 was considered statistically significant.

## Results

### Validation of consistency of CAF algorithms and identification of CAF as an adverse prognostic factor in HGSOC

**Figure 1:**
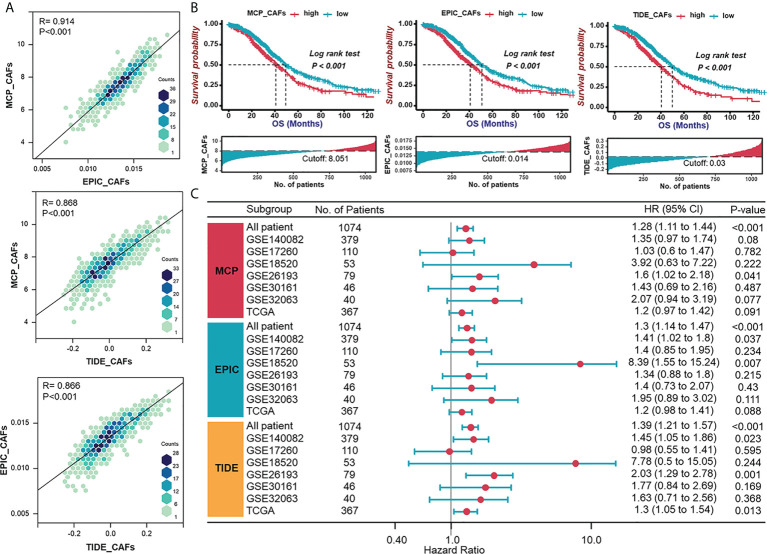
Validation of consistency of CAF algorithms and identification of CAF as an adverse prognostic factor in HGSOC. **(A)** The correlation between any two signatures obtained by three CAF algorithms (MCP, EPIC, and TIDE) in each patient in the integrated cohort (TCGA-OV and GEO datasets). **(B)** Survival analyses for patient with relatively high or low CAF signatures in the integrated cohort using Kaplan–Meier curves. **(C)** Forest plot of univariate Cox analysis of CAF signatures in TCGA and GEO datasets.

To examine whether CAF signatures calculated by three recently established algorithms (MCP-CAFs, EPIC-CAFs, and TIDE-CAFs) are generally consistent and stable, we evaluated the correlation between any two signatures from the three algorithms in each HGSOC patient from the integrated cohort (**Figure 1A**). The strong positive correlation between each signature is reflected by R=0.914, 0.868, and 0.866, respectively. In order to characterize CAFs comprehensively and convincingly, all three algorithms would be applied simultaneously during the subsequent analysis. HGSOC patients were grouped according to the content of CAFs, and significant prognostic differences were observed between groups (**Figure 1B**). Further, univariate Cox regression analysis determined CAF signatures as indexes suggesting adverse prognosis (**Figure 1C**).

### Investigating the relevance between CAF signatures and components as well as signals in HGSOC immune microenvironment

**Figure 2:**
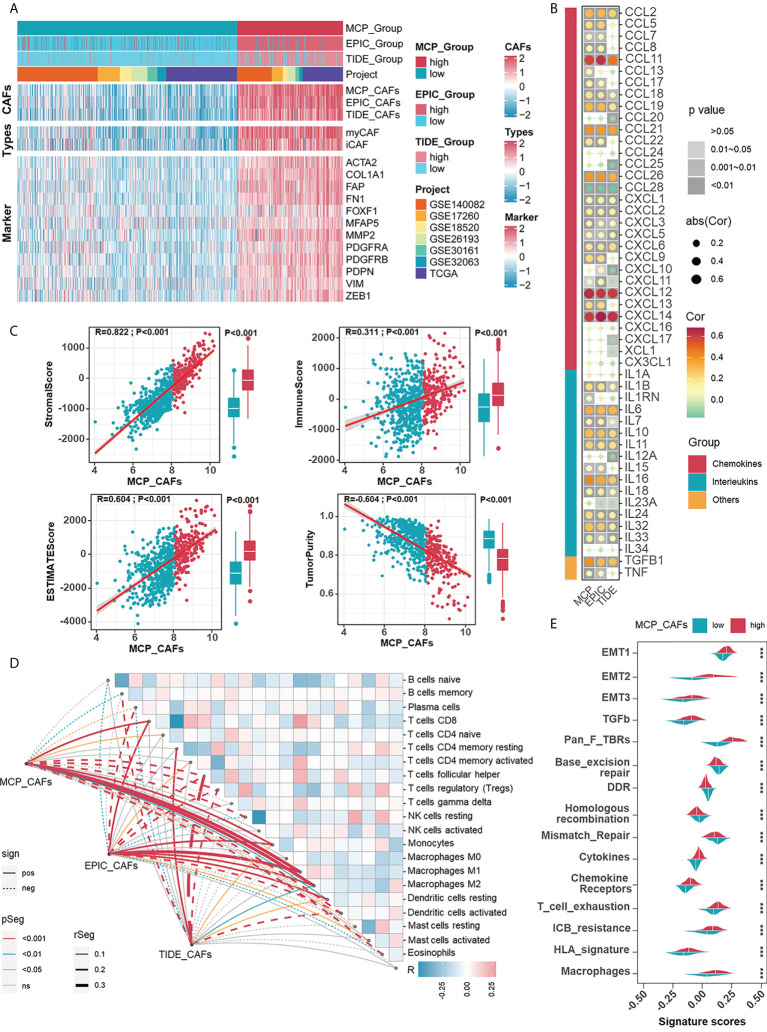
Investigating the relevance between CAF signatures and components as well as signals in HGSOC immune microenvironment. **(A)** The heatmap shows the correlations between CAF signatures and acknowledged makers of CAFs in 1074 HGSOC patients in meta cohort. Pink represented the relatively high score or expression and blue represented the relatively low score or expression. **(B)** Correlations between CAF signatures with cytokines including chemokines, interleukins, and other cytokines. **(C)** Correlations between CAF signatures (MCP) and ImmuneScore, StromalScore, ESTIMATEScore as well as Tumor Purity. **(D)** Correlations between CAF signatures (MCP) and immune cells (cibersort). **(E)** Differences in ICB response-related signatures between low- and high-CAF (MCP) groups. ***, P < 0.001.

Given the impressive ability of CAF signatures to distinguish between patients with widely varying outcomes, the role of CAFs in the prognosis of HGSOC patients deserves further exploration. The relevance of CAFs to prognosis should be demonstrated by more proof. Specifically, CAF algorithms and acknowledged sub-CAF signatures (myofibroblastic CAFs, myCAFs; inflammatory CAFs, iCAFs), as well as markers, are supposed to measure the content of CAFs. Prognostic-oriented clustering in the HGSOC integrated cohort exhibited distinct CAF signatures as well as markers between the two groups (**Figure 2A**). As a prominent population in the TME of HGSOC, CAFs undeniably engage in shaping the immune microenvironment. Thus, we demonstrated correlations between CAF signatures with cytokines including interleukins and chemokines, in which CCL11, CXCL12, and CXCL14 were remarkable because of their apparent positive correlations with CAFs (**Figure 2B**). Based on the CAF signature displayed by the MCP algorithm, the ESTIMATE algorithm showed that CAFs were inversely correlated with Tumor Purity but positively correlated with the ImmuneScore, StromalScore, and ESTIMATEScore (**Figure 2C**). Additionally, CAF signatures positively correlated with macrophage abundance (**Figure 2D**), enhanced immunotherapy resistance, and mesenchymal activation, including epithelial-mesenchymal transition (EMT), TGF-β signals, and pan-fibroblast TGF-β response (Pan-F-TBRS) in the high CAFs group (**Figure 2E**). Heretofore, the adverse role of CAFs in HGSOC was reflected by not only the overall survival of patients but also possible participation in mesenchyme activation, immunosuppression, and resistance to immunotherapy.

### Detection and functional interpretations of a gene co-expression module shared CAF characteristics in HGSOC

**Figure 3:**
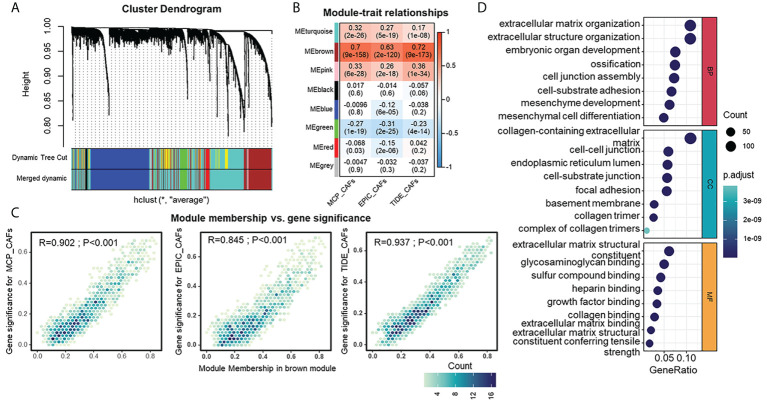
Detection and functional interpretations of a gene co-expression module shared CAFs characteristics in HGSOC. **(A)** Network analysis of gene expression in HGSOC cohort. Dendrograms obtained by average linkage hierarchical clustering of genes on the basis of topological overlap. Modules of co-expressed genes were assigned colors. The color row underneath the dendrogram shows the modules assigned by the Dynamic Tree Cut and merged to produce eight distinct modules. **(B)** Module-trait relationships: Each column corresponds to a trait, and each row corresponds to a module eigengene. The number in the rectangle indicates the correlation coefficients (P-values in the brackets). The table is color-coded by correlation based on the color legend: red to blue indicates a positive to negative correlation of module eigengenes with traits. **(C)** Scatter plot of module eigengenes in brown module. **(D)** Gene Ontology analysis of genes in the brown module. CC, cellular component (upper); MF, molecular function (middle); BP, biological process (bottom).

Identification of genes that show similar expression patterns across samples might help shed light on shared biological processes, for example, the mechanism for activation of CAFs in TME. Thus, we investigated this by applying WGCNA to an integrated HGSOC cohort generated from TCGA and GEO datasets. β=3 was selected to construct a standard scale-free network with the pick soft threshold function (**Figure S1A**), where genes were assigned to eight different modules using a cluster dendrogram (**Figure 3A**). To identify the module regulating CAFs, we correlated each module eigengene with different CAF traits, suggesting the brown module’s potential. The full module-trait correlation table is presented in **Figure 3B**. The brown module members present good correlations with CAF signatures (**Figure 3C**). Furthermore, GO analysis revealed that brown module genes were mainly enriched in functions such as extracellular matrix organization, collagen-containing extracellular matrix, and extracellular matrix structural constituents (**Figure 3D**). KEGG analysis of brown module genes emphasized the PI3K-Akt signaling pathway, focal adhesion, and ECM-receptor interaction (**Figure S1B**). The above results raise the possibility that the brown module is a specific gene network regulating the ECM and sharing similarities with the CAF traits of HGSOC.

### The hub genes extracted from CAFs-related module as potential CAF markers participating in the shaping of TME

**Figure 4:**
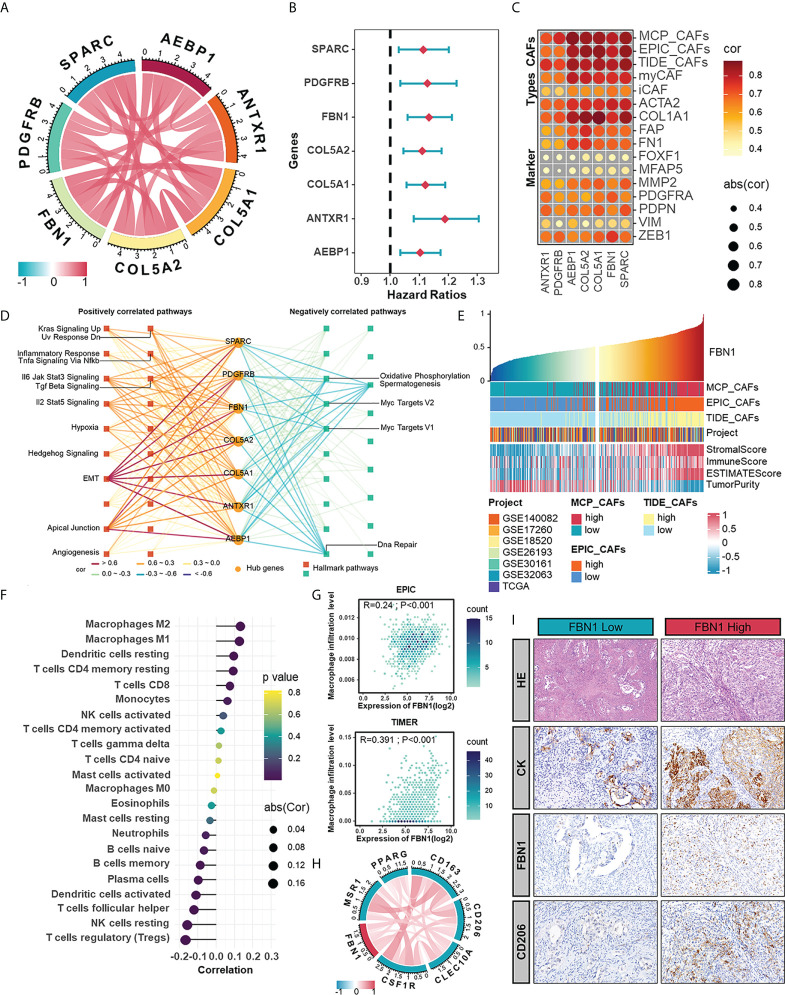
The hub genes extracted from CAFs-related module as potential CAF markers participating in the shaping of tumor microenvironment. **(A)** The correlations between any two hub genes. **(B)** Forest plot of univariate Cox analysis of hub genes in meta-cohort. **(C)** The correlations between hub genes and CAF signatures (MCP, EPIC, and TIDE) and CAF markers. **(D)** Hallmark pathways in which hub genes were involved. **(E)** The TME scores in HGSOC samples ranked by the mRNA level of FBN1. **(F)** The correlation between the mRNA expression of FBN1 and the level of immune cell infiltration calculated by CIBERSORT. **(G)** The positive correlations between the mRNA expression of FBN1 and the level of macrophage infiltration calculated by EPIC and TIMER. **(H)** The correlations between the expression of FBN1 and M2 macrophage markers in mRNA levels. **(I)** HE staining and IHC staining of CK, FBN1 and CD206 in one sample with relatively low protein expression of FBN1 and the other with high protein expression of FBN1.

Highly connected “hub” genes are thought to be paramount in managing the behavior of biological modules (39). Therefore, we set out to identify the hub genes in the brown module and hypothesized that those hub genes might be associated with CAFs in HGSOC. The relationships between genes in the brown module and CAFs signatures were evaluated with GS and MM. In the end, seven hub genes, including anthrax toxin receptor 1 (ANTXR1), pericytes derived growth factor receptor beta (PDGFRB), adipocyte enhancer-binding protein 1 (AEBP1), collagen type V alpha 2 chain (COL5A2), collagen type V alpha 1 chain (COL5A1), FBN1, and secreted protein acidic and cysteine rich (SPARC), were screened out with GS >0.3 and MM >0.8 (**Figure 4A**). Among them, PDGFRB is an acknowledged marker of CAFs (40), which supports the accuracy of our results and suggests the potential of other genes to characterize the abundance or properties of CAFs. Univariate regression analysis confirmed the adverse role of hub genes in the prognosis of HGSOC (**Figure 4B**). Significant functions of these hub genes in modulating biological processes relating to CAFs were validated by fair positive correlations between hub genes and signatures as well as markers of CAFs (**Figure 4C**). To extend other biological features from hub genes themselves, we performed GSVA enrichment analysis and revealed strong positive correlations between hub genes with mesenchymal activation and classic cancer-promoting pathways such as TGF-β signaling pathway, EMT, apical junction, and angiogenesis (**Figure 4D**).

In the further investigation of relationships between hub genes and the immune microenvironment of HGSOC, we focused on FBN1, an ECM glycoprotein that has been reported to promote the structure formation of calcium-binding microfibrils (41). The ESTIMATE algorithm was performed to assess components in the TME of patient samples ranked by the mRNA level of FBN1. Those patients with relatively high FBN1 expression also owned higher StromalScore and ImmuneScore (**Figure 4E**). As for specific immune components, FBN1 was positively correlated with the infiltration of subpopulations of T cells and myeloid cells, especially macrophages, which was further verified by the EPIC and TIMER methods (**Figures 4F, G**). Also, there were positive correlations between FBN1 and biomarkers of immunosuppressive macrophages as well as some immune checkpoints (**Figures 4H**, **S2A**). The preference of the FBN1 expression in fibroblasts was confirm in a single cell RNA set (GSE118828) downloaded from the Tumor Immune Single-cell Hub (TISCH) (**Figures S2B-D**). Immunohistochemical staining further verified that FBN1 was mainly expressed in mesenchymal cells, and patients with relatively high FBN1 expression also had a higher positive rate of CD206 (a marker of immunosuppressive macrophages) (**Figure 4I**). From the above results, we could speculate that CAFs expressing FBN1 may be involved in the formation and maintenance of the immunosuppressive microenvironment of HGSOC. The morphological distribution and approximate protein levels of other hub genes that existed in The Human Protein Atlas and TISCH are visualized in **Figures S3A-C**. Besides, each hub gene was also assessed on its correlations with scores calculated by the ESTIMATE algorithm and the enrichment scores of various types of immune cells (**Figure S4A**).

### Establishment of the CAFscore evaluation system as a prognostic indicator in HGSOC and its involvement in TME

**Figure 5:**
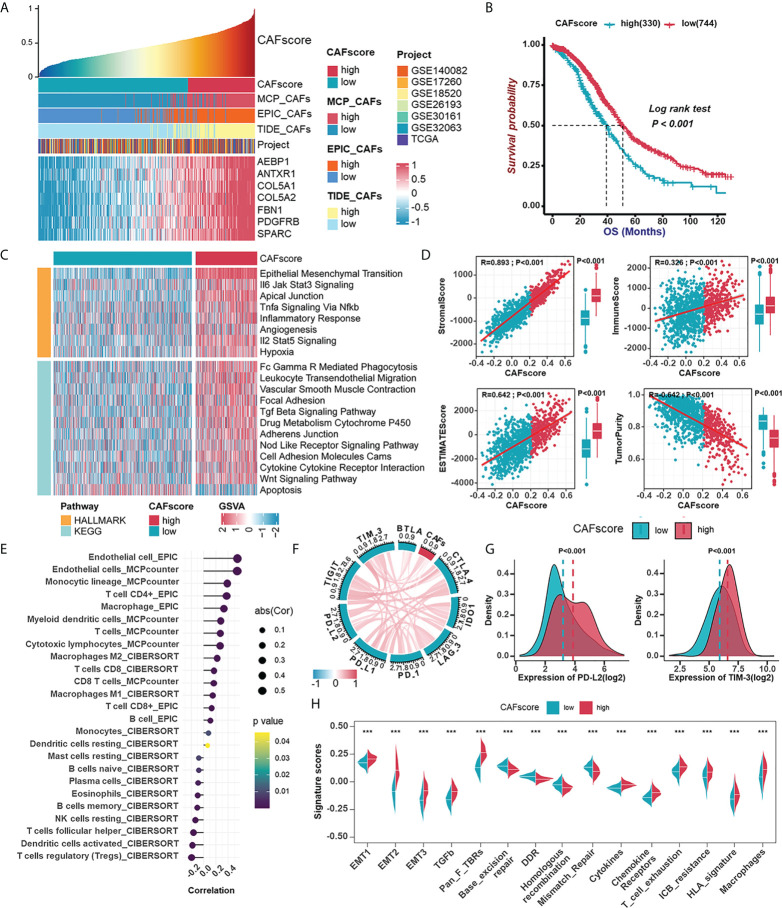
Establishment of the CAFscore evaluation system as a prognostic indicator in HGSOC and its involvement with immune microenvironment. **(A)** The correlations between the mRNA expression of hub genes and CAFscore. **(B)** Survival analyses for patient with relatively high- or low-CAFscore in the integrated cohort using Kaplan–Meier curves. **(C)** GSVA enrichment analysis showing the activation states of biological pathways in high- and low-CAFscore group. The heatmap was used to visualize these biological processes, and yellow represented HALLMARK database and blue represented KEGG database. **(D)** The correlations between CAFscore and TME scores. **(E)** The correlations between the CAFscore and the level of immune cell infiltration. **(F)** The correlations between the CAFscore and the expression of ICMs (CTLA4, IDO-1, LAG-3, PD-1, PD-L1, PD-L2, TIGIT, TIM-3, BYLA). **(G)** Comparisons of PD-L2 (left) and TIM-3 (right) expression levels between high- and low-CAFscore groups. **(H)** The differences in the enrichment scores of stroma-activated pathways, DNA damage repair pathways and ICB response-related signatures between high- and low-CAFscore groups. ***, P < 0.001.

Considering the significant impact of CAF traits on prognosis, we picked out 145 genes in the brown module when setting GS >0.3 and MM >0.6 to construct a scoring system which was termed CAFscore, using ssGSEA for measuring the prognosis of HGSOC patients. These 145 genes were enriched in extracellular matrix organization and mesenchymal activation, as revealed by GO and KEGG analyses, similar to the enriched pathways of the entire brown module (**Figures S5A, B**). Besides, the CAFscore correlated well with hub genes and CAF signatures (**Figure 5A**). Meanwhile, HGSOC patients were divided into a high CAFscore group (n=330) and a low CAFscore group (n=744) based on an optimal cutoff value for the CAFscore. Kaplan-Meier analyses and univariate regression analysis suggested the CAFscore was a prominently adverse prognostic factor in the integrated cohort and most HGSOC patient datasets with sufficient samples (**Figures 5B**, **S6A, B**). Particularly, in the TCGA-OV cohort, multivariate Cox regression analysis revealed that the CAFscore was an independent prognostic factor (**Figure S6C**). In the pan-cancer univariate regression analysis, the CAFscore as a risk factor was also applicable to most cancer types (**Figure S6D**). The heatmap in **Figure 5C** demonstrated the enhanced EMT and inflammatory signaling pathways (“IL6-JAK-STAT3 signaling”, “TNFα signaling *via* NF-κb”, “inflammatory response”, and “IL2-STAT5 signaling”), the release of cytokines (“TGF-β signaling pathway” and “cytokine-cytokine receptor interaction”), activated immunoreaction (“Fc Gamma R mediated phagocytosis” and “leukocyte transendothelial migration”), and impaired “apoptosis” in the high CAFscore group. The CAFscore evaluation system was used to create a landscape of TME characteristics and immune cell infiltration, which revealed that the CAFscore was positively correlated with not only the StromalScore and ImmuneScore (**Figure 5D**) but also the infiltration of endothelial cells, myeloid cells, and CD4+ T cells (**Figure 5E**), particularly immunosuppressed macrophages (**Figure S7A**). Also, the CAFscore, it should be noted, is well related to the expression of immune checkpoints, especially PD-L2 and TIM-3 (**Figures 5F, G**). Paramount biological pathways engaging in the tumor immune microenvironment were evaluated in high- and low-CAFscore groups. Among them, signatures related to immunotherapy resistance and mesenchymal activation were enriched, but the DNA damage repair pathway was impaired in the high CAFscore group (**Figure 5H**). The above results suggest the capacity of the CAFscore for indicating individual prognosis and the ability of CAFs to form the immunosuppressive and stroma-activated TME of HGSOC.

### The role of the CAFscore in the prediction of immunotherapy benefits and the selection of sensitive chemotherapeutic agents

**Figure 6:**
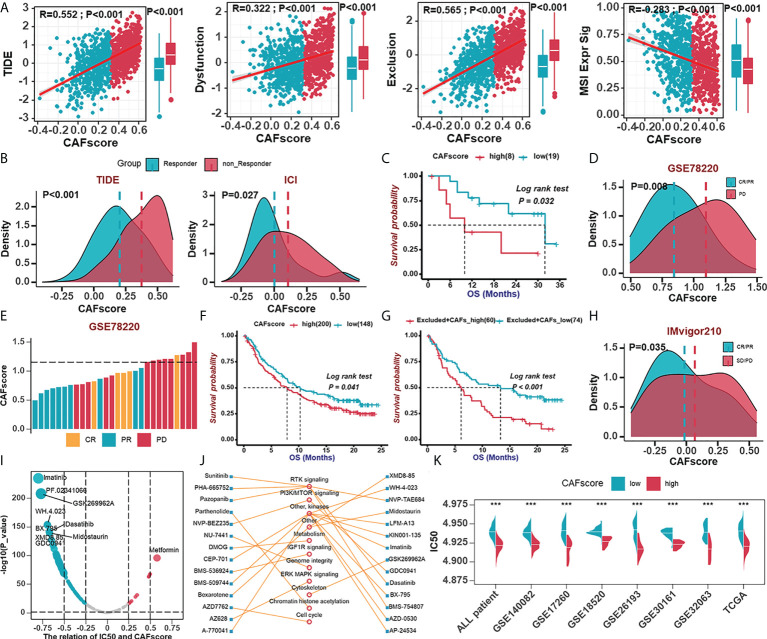
The role of the CAFscore in the prediction of immunotherapy benefits and the selection of sensitive chemotherapeutic agents. **(A)** The correlations between CAFscore and TIDE, Dysfunction, Exclusion, and MSI expression signature. **(B)** The comparison of CAFscores between responder and non-responder groups, according to TIDE (left) and ImmuCellAI (right) algorithms. **(C)** Survival analyses for low (19 cases) and high (8 cases) CAFscore patient groups in the GSE78220 cohort using Kaplan–Meier curves. **(D)** The CAFscore in the group with complete response (CR) or partial response (PR) versus the group with progressive disease (PD) in GSE78220. **(E)** The association of the CAFscore with clinical response to anti-PD-1 immunotherapy per patient in GSE78220 cohort. **(F)** Survival analyses for low (148 cases) and high (200 cases) CAFscore patient groups in IMvigor210 cohort using Kaplan–Meier curves. **(G)** Survival analyses for low (74 cases) and high (60 cases) CAFscore patient groups in the excluded immune subgroup. **(H)** The CAFscore in the group with CR/PR versus the group with PD/stable disease (SD) in IMvigor210 cohort. **(I)** The correlations between CAFscore and the estimated IC_50_ for drugs evaluated by the Spearman analysis. Each point represents a drug. **(J)** The lines represent the relationship between candidate drugs and pathways. **(K)** The differences in the estimated IC_50_ for lmatinib between high- and low-CAFscore groups in TCGA and GEO datasets, respectively. ***, P < 0.001.

To further elucidate the effects of CAFscore in the context of immunotherapy (represented by ICBs), we first extended our analysis to associations between CAFscore and tumor mutation burden (TMB), which may influence cancer immunogenicity. In the TCGA-OV cohort, there were limited correlations between CAFscore and mutation counts and a less significant difference in TMB across CAF groups (**Figure S8A**). To predict ICB response, newly identified predictors, such as TIDE scores and ImmuCellA (**Figures 6A, B**, S8B), are widely used to evaluate the immune response (30, 38). The CAFscore was positively correlated with TIDE, dysfunction, and exclusion and negatively correlated with MSI Expr sig, and the CAFscore of HGSOC patients who responded to immunotherapy was lower than those who did not. Next, assessing the ability of the CAFscore to predict patients’ responses to ICB in both immunotherapy cohorts: GSE78220 (**Figures 6C–E**) and IMvigor210 (**Figures 6F–H**) revealed that survival benefits and response to ICB treatment were observed in patients with a low CAFscore (**Figures 6C–H**, **S8C, D**). In the IMvigor210 cohort, patients were divided into deserted, excluded, and inflamed subgroups based on the infiltration status of CD8+ T cells (42). In different immune phenotype subgroups, the overall survival of patients and responses to ICB treatment varied based on the CAFscore system. For example, a high CAFscore represents poor prognosis and resistance to ICB in the excluded subgroups (**Figure 6G**, **S8E**) but not in the deserted subgroup (**Figure S8C**). This suggests that different levels of activation or infiltration of CAFs result in different degrees of immunosuppression if there are immune components, thus leading to variations in resistance to immunotherapy and the outcome of patients.

To understand the effect of CAFscore on the clinical efficacy of HGSOC treatments, we analyzed correlations between CAFscore and IC_50_ of drug candidates in the Genomics of Drug Sensitivity in Cancer (GDSC) database (**Figure 6I**). A total of 31 drug candidates with |*Rs*| >0.5 were screened out, the IC_50_ of the most of which (30 candidates) inversely correlated with CAFscore, targeting PI3K-mTOR signaling, RTK signaling, and other kinases (**Figure 6J**). Remarkably, the estimated IC_50_ of imatinib exerted a pretty negative correlation with the CAFscore, which means this agent might benefit patients with a high CAFscore (**Figure 6K**). Together, these results implied that CAFs played crucial roles in mediating the immune response and correlated with drug sensitivity. Thus, the CAFscore might be a potential biomarker for establishing appropriate treatment strategies.

## Discussion

Tumor stroma and the immune microenvironment have received an extensive concern for nearly a decade. The effector cells that can kill tumor cells always garner much attention in the development of immunotherapy. Nevertheless, the limited benefit to HGSOC patients from ICB drives researchers to investigate the mechanisms of this resistance and other novel targets. As early as 2010, TCGA termed four HGSOC subtypes based on gene content, of which the mesenchymal subtype was characterized by high expression of HOX genes and increased stromal components such as for myofibroblasts and microvascular pericytes (4). Also, the mesenchymal subtype was identified by several independent studies. Thus, targeting CAFs, a major component of the stroma, by altering their numbers, subtypes, or functionality, is being explored as an avenue to improve cancer therapies. In this study, we aim to evaluate the effect of CAFs on the prognosis and response to immunotherapy of HGSOC patients.

Several independent CAF signatures, as well as some well-known fibroblast markers, including fibroblast activation protein alpha (FAP), collagen 1A1 (COL1A1), and platelet-derived growth factor receptor alpha (PDGFRA), were employed to evaluate the content of CAFs. Kaplan-Meier survival estimation and univariate Cox regression analysis confirmed that CAF could be a risk factor for HGSOC patients, further verified by prognostic-oriented clustering of HGSOC samples.

As a substantial source of growth factors and cytokines, CAFs certainly orchestrate the composition and content of soluble substances in TME and thus contribute to tumor progression. The correlations between CAF signatures and components of the immune microenvironment as well as ICB-related signatures implied that elevated expression of cytokines (e.g., CCL11, CCL21, CXCL12, CXCL14, IL-6, IL-16, and TGF-β), the enrichment of immunosuppressive macrophages, mesenchymal activation, and enhanced immunotherapy resistance were all linked to CAFs-enriched microenvironment. There have been specific studies about CXCL12 and CXCL14 in CAFs in tumor progression. Fibroblast-derived CXCL12 facilitated tumor cell intravasation and limited T cell-mediated tumor control (42–44). Also, CAFs expressed CXCL14 for their tumor-supporting properties (45, 46). Besides, it is not known whether CCL11 (a chemokine for eosinophils, engaging in ovarian cancer progression) (10, 47), CCL21 (a chemokine for thymocytes and activated T cells, mediating homing of lymphocytes) and IL-16 (a modulator of T cell activation) participate in the functionality of CAFs and the remodeling of the tumor immune microenvironment, which might deserve further exploration. What is noteworthy is that enhanced expressions of IL-6 and TGF-β are linked. Experiments and observations show that iCAFs express less ACTA2 (actin alpha2 smooth muscle, also abbreviated as -SMA) and secrete more IL-6 and other inflammatory factors (e.g., IL-8, IL-11, CXCL1, and CXCL2), thereby participating in immune suppression, whereas myCAFs are responsible for ECM remodeling, with high TGF-β and ECM proteins such as fibronectin 1, and COL1A1 (48, 49). Given the relatively weaker expression of iCAF markers than ECM components and myCAF markers in CAFs-enriched samples, we suppose that myofibroblasts are predominant CAF population in HGSOC. As regards macrophages, CAFs-enriched environments also exhibited high expression of CCL2 (also named monocyte chemoattractant protein-1, MCP-1), which might partly explain the abundance of macrophages.

For a deeper understanding of the potential molecular mechanisms of the link between CAFs and prognosis in HGSOC patients, we performed WGCNA to find modules of highly correlated genes and correlated modules to CAF traits, and focused on the brown module that was characterized by enrichment pathways and GO groups defining ECM production and remodeling, cell adhesion, and angiogenesis. Afterward, we extracted intramodular hub genes from the brown module that exhibit excellently positive correlations with CAF traits. These hub genes, conceivably, contain markers of collagen (COL5A2 and COL5A1) and PDGFRB. To identify a potential novel gene target, we looked for highly connected genes that have not been extensively studied as cancer targets and then focused on FBN1, a structural component of calcium-binding microfibrils. A few studies reveal that FBN1 is a risk factor for hematogenous and lymphatic metastasis in serous ovarian cancer and promotes chemoresistance in ovarian cancer organoids (50, 51). The predominance of FBN1 in fibroblasts were confirmed in a single cell RNA set (GSE118828) downloaded from the TISCH database (52). Based on the contribution of CAFs, we hypothesize that FBN1 plays an adverse role in HGSOC prognosis.

In addition to the contribution to tumor growth, CAFs also influence the infiltration and properties of other tumor microenvironment components, which greatly accounts for resistance to ICB. All signatures, from CAF algorithms to hub genes in CAF-related modules to the CAFscore, indicate the importance of macrophages and T leukocytes in the immune microenvironment remolded by CAFs. In detail, CAFs advanced the recruitment of monocytes (macrophage precursors) and their differentiation into immunosuppressive macrophages (usually referred to as M2 macrophages) *via* multiple regulatory molecules, including macrophage colony-stimulating factor 1 (M-CSF1), IL-6, CCL2, and TGF-β, thereby impairing responses from effector T cells and inducing immune suppression in the TME (53–57). In turn, M2 macrophages were also able to enhance EMT to stimulate activation of CAFs and influence the trans-differentiation and activity of mesenchymal stem cells (one of the cellular precursors of CAFs) (58). Besides, the relevance between immunosuppressive macrophages and CAFs was confirmed by IHC staining of CD206 and FBN1 in our study, further supporting the adverse role of FBN1 in HGSOC. Numerous studies have illustrated the role of CAFs in modulating T cell activities and functions. CAFs can recruit CD8+ cytotoxic T cells. A study based on the single-cell dissection of cellular components in ovarian cancer revealed that CAFs-T cells cross-talk relies on the CXCL12/14-CXCR4 axis (59). However, immunosuppression is a general feature of the TME of HGSOC, which is consistent with the result that CAF signatures and the CAFscore are positively correlated with T cell exhaustion as well as dysfunction and resistance to ICB. Also, CAFs stimulate the migratory activity of Treg cells and markedly increase their frequency in colorectal tumor sites, and promote Th2 polarization in pancreatic cancer (60, 61). Furthermore, CAFs exerted immunosuppressive effects from the following several aspects: modulating the degree of tumor-associated neutrophils (TANs) activation (62); cooperating with mast cells to induce the early malignant morphological transition of benign epithelial cells (63); impairing the functionality of infiltrating NK cells (64, 65); blocking DC maturation and antigen presentation (18); increasing attraction and differentiation of Tregs (66).

The CAFscore was contracted to indicate the relative content of CAFs in individuals, which was further suggested as an independent prognostic factor by univariate Cox regression analysis, not only in HGSOC samples but also in pan-cancer. HGSOC samples with high CAFscore exhibited poor overall survival and enrichment of immune and inflammatory pathways, cell connection and adhesion, and angiogenesis, which inspired us to explore the potential of the CAFscore in predicting the response to immunotherapy in HGSOC. Prior studies indicate that cancers with high TMB are more likely to benefit from ICBs (67). However, there were limited correlations between CAFscore and mutation counts and less significant differences in TMB across CAF groups, which could be explained as extensive cross-talk between CAFs and other ICB response determinants, such as mesenchymal activation and immunosuppressive TME. HGSOC samples with high CAFscore had significant stroma activation status (including the highly expressed EMT and TGF-β pathways, as well as Pan-F-TBRS), as well as increased infiltration of immunosuppressive cells and ICM expression. HGSOC patients with higher CAFscore not only tended not to respond to ICBs but also were more prone to immune escape when using TIDE and ImmuCellA to evaluate the immune response. Further, survival benefits and response to ICB treatment were observed in a patient with a low CAFscore from two anti-PD-1 immunotherapy cohorts. In the IMvigor210 cohort, when patients were divided into deserted, excluded, and inflamed subgroups based on the infiltration status of CD8+ T cells (42), high CAFscore represented poor prognosis and resistance to ICB in the excluded subgroup, but not in the deserted subgroup. Tumors with immune excluded phenotypes have an abundance of immune cells retained in the stroma rather than penetrating the tumor mass. The activation of stroma in TME was thought to suppress T cells (68). The existence of CD8+ T cells enables CAFs to exert their influence on shaping the immune microenvironment. Different levels of activation and infiltration of CAFs result in different degrees of immunosuppression if there are immune components, thus leading to variations in resistance to immunotherapy and the outcome for patients. Besides, more potential drug treatments were adapted in the high CAFscore group in the drug sensitivity analysis, suggesting another treatment strategy.

There are still many deficiencies in this study that should be paid attention to and further explored. Firstly, we retrospectively construct the CAFscore based on public datasets and patient samples of our own, but complete clinical parameters alone are not sufficient to support our prognosis model. Secondly, HGSOC patients are divided into two groups just based on the content rather than the different properties of CAFs. Finally, prospective cohorts of HGSOC patients receiving immunotherapy are needed to validate our findings further.

In conclusion, this study provides a valuable tool to evaluate the content of CAFs from gene expression data, which is represented as the CAFscore, with the properties of predicting HGSOC patient prognosis and revealing the degree of immunosuppression. Individualized assessment of CAFscore informs therapeutic strategies to improve clinical benefit from cancer therapies.

## Data availability statement

The original contributions presented in the study are included in the article/**Supplementary Material**. Further inquiries can be directed to the corresponding authors.

## Ethics statement

The study was reviewed and approved by Ethics Committee of Xiangya Hospital Central South University, approval number 202204081. The ethics committee waived the requirement of written informed consent for participation.

## Author contributions

Conceptualization, YL and RT; formal analysis, YL; funding acquisition, JLi; investigation, RT; methodology, RT and YL; project administration, QW; supervision, XF; visualization, HT; writing—original draft, RT; writing—review and editing, JLiu and YL. All authors have read and agreed to the published version of the manuscript.

## Funding

This work was supported by National Natural Science Foundation of China (Grant No.82103300); Natural Science Foundation of Hunan Province of China (Project No. 2019JJ50857).

## Conflict of interest

The authors declare that the research was conducted in the absence of any commercial or financial relationships that could be construed as a potential conflict of interest.

## Publisher’s note

All claims expressed in this article are solely those of the authors and do not necessarily represent those of their affiliated organizations, or those of the publisher, the editors and the reviewers. Any product that may be evaluated in this article, or claim that may be made by its manufacturer, is not guaranteed or endorsed by the publisher.

## Supplementary material

Supplementary Figure 1 Detection and functional interpretations of a gene co-expression module shared CAFs characteristics in HGSOC. **(A)** Left: An examination of the scale-free fit index for a variety of soft-threshold values (β); Right: An analysis of the mean connectedness for different soft-threshold values. **(B)** KEGG pathway analysis of genes in the brown module.

Supplementary Figure 2 The correlations between the expression of FBN1 and immune microenvironment **(A)** The correlations between the expression of FBN1 and ICMs in mRNA levels. **(B)** Scatter plot showing the cell clusters in GSE118828. **(C-D)** Scatter plot **(C)** and violin plot **(D)** showing the distribution of cells expressing a high level of FBN1 in GSE118828.

Supplementary Figure 3 The hub genes extracted from CAFs-related module as potential CAFs markers participating in the shaping of immune microenvironment. **(A, B)** Scatter plot **(A)** and violin plot **(B)** showing the expression of other hub genes for all cell types in GSE118828. **(C)** The morphological distribution and approximate protein levels of other hub genes.

Supplementary Figure 4 The correlations between hub genes and immune cells.

Supplementary Figure 5 Enrichment analyses of CAFs-related genes**. (A, B)** GO pathways **(A)** and KEGG pathways **(B)** in which genes constructing CAFscore were enriched.

Supplementary Figure 6 The CAFscore is a prognostic indicator in HGSOC**. (A)** Survival analyses for patient with relatively high- or low-CAFscore in GEO database (GSE140082, GSE18520, GSE26193) and TCGA-OV database using Kaplan–Meier curves. **(B)** Univariate Cox regression analyses estimating prognostic value of the CAFscore in different HGSOC cohorts. **(C)** Univariate and multivariate Cox regression analyses of the CAFscore with age, tumor grade and stage in the TCGA-OV cohort. HR and p-values were displayed. **(D)** Univariate Cox regression analyses estimating prognostic value of the CAFscore in different cancer types from TCGA dataset.

Supplementary Figure 7 The correlations between the CAFscore with the expression of immunosuppressive macrophage markers in mRNA levels.

Supplementary Figure 8 The role of the CAFscore in the prediction of immunotherapy benefits **(A)** The correlations between the CAFscore and synonymous mutation counts, non-synonymous mutation counts, and all mutation counts in TCGA-OV cohort. **(B)** The comparison of CAFscores between Responder and non-Responder groups in TCGA and GEO datasets, respectively, according to TIDE algorithms. *, P < 0.05; **, P < 0.01; ***, P < 0.001. **(C, D)** Survival analyses for low and high CAFscore patient groups in the desert **(C)** and inflamed **(D)** immune subgroups. **(E)** The CAFscore in the group with CR/PR versus the group with PD/(SD) in the desert (left), excluded (middle) and inflamed (right) immune subgroups.
