# Supplementary figures and images for: Deciphering the immune landscape dominated by cancer-associated fibroblasts to investigate their potential in indicating prognosis and guiding therapeutic regimens in high grade serous ovarian carcinoma
Source: Front Immunol. 2022 Sep 2;13:940801. doi: 10.3389/fimmu.2022.940801 (PMC9478207; doi:10.3389/fimmu.2022.940801)

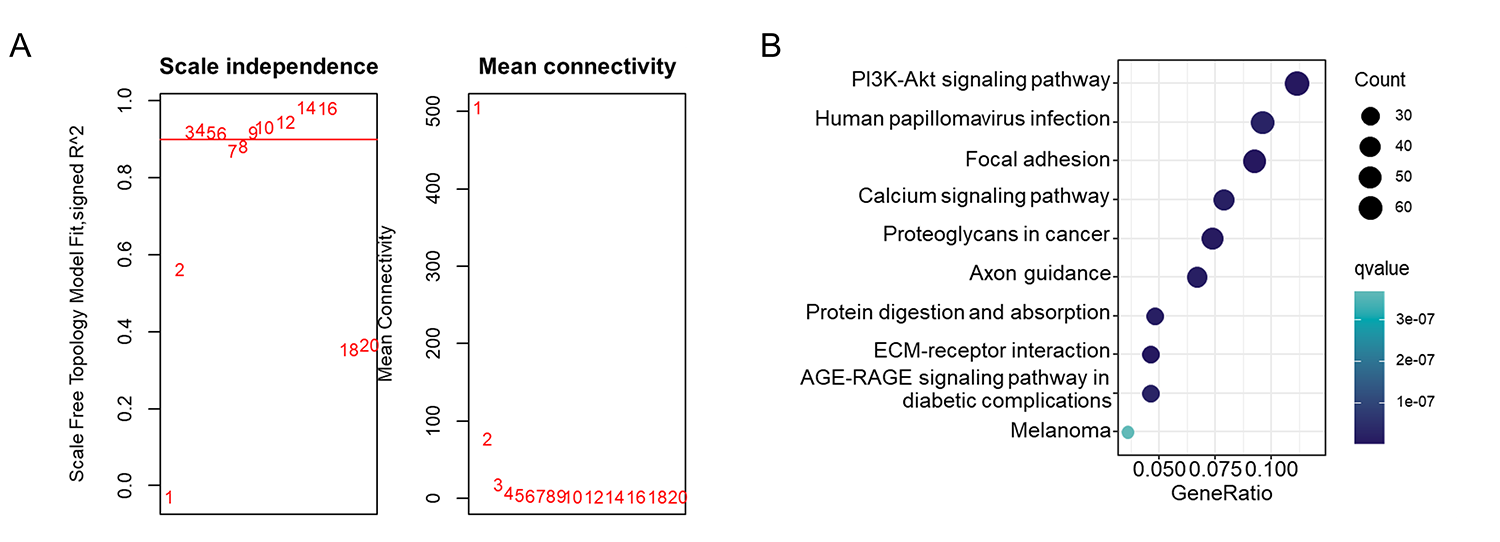

Supplement: Supplementary Figure 1 — Detection and functional interpretations of a gene co-expression module shared CAFs characteristics in HGSOC. (A) Left: An examination of the scale-free fit index for a variety of soft-threshold values (β); Right: An analysis of the mean connectedness for different soft-threshold values. (B) KEGG pathway analysis of genes in the brown module. [file Image_1.tif]

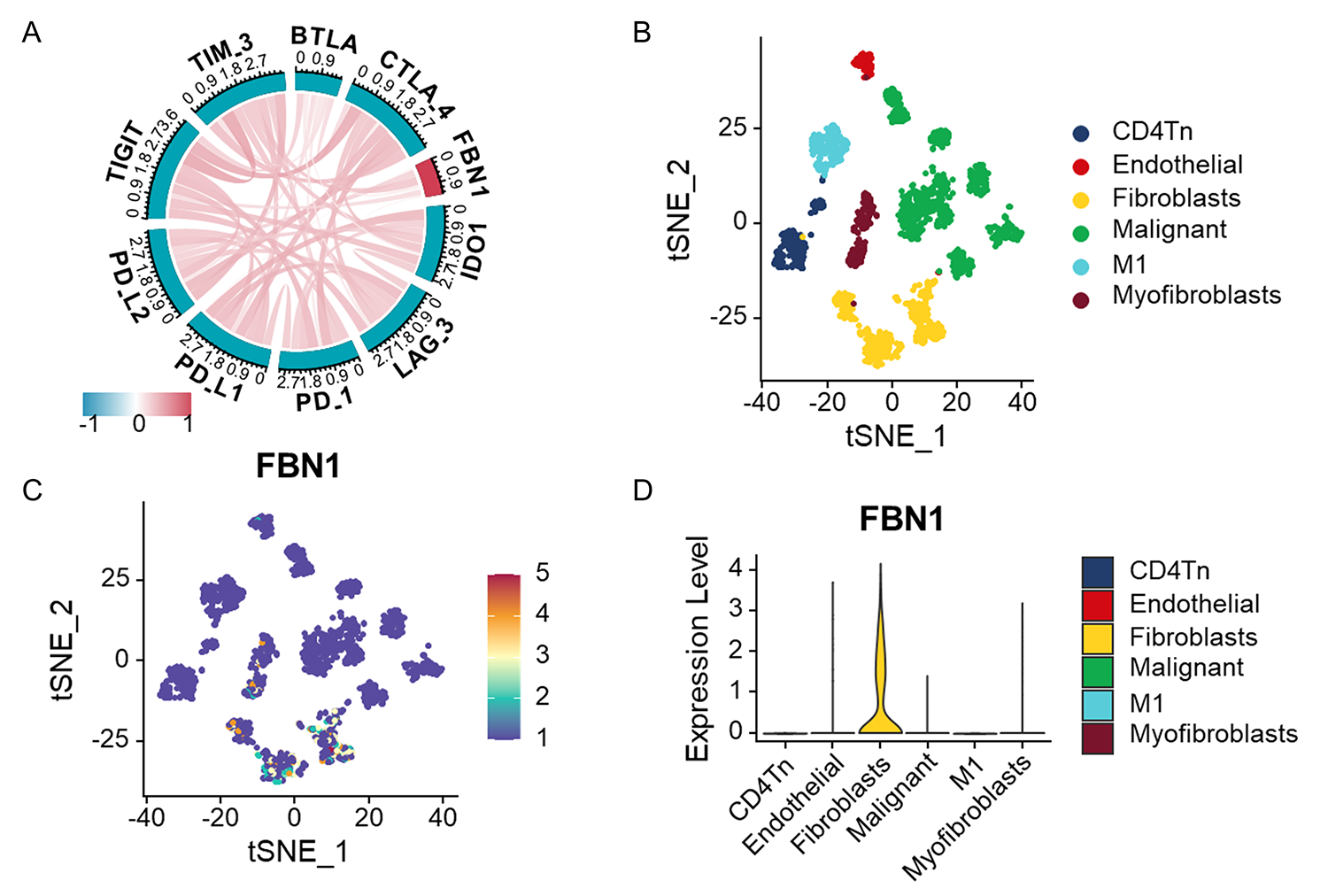

Supplement: Supplementary Figure 2 — The correlations between the expression of FBN1 and immune microenvironment (A) The correlations between the expression of FBN1 and ICMs in mRNA levels. (B) Scatter plot showing the cell clusters in GSE118828. (C-D) Scatter plot (C) and violin plot (D) showing the distribution of cells expressing a high level of FBN1 in GSE118828. [file Image_2.tif]

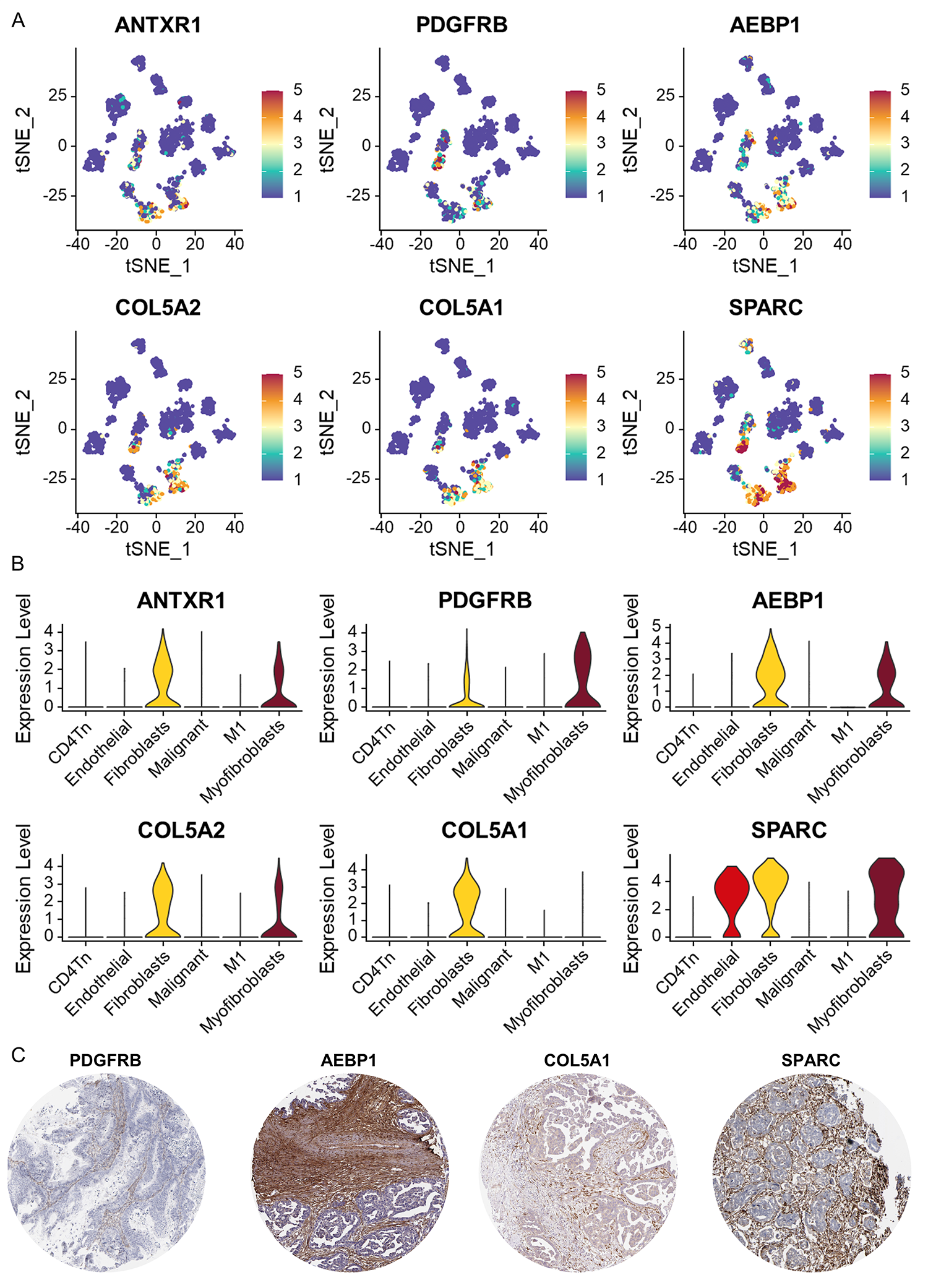

Supplement: Supplementary Figure 3 — The hub genes extracted from CAFs-related module as potential CAFs markers participating in the shaping of immune microenvironment. (A, B) Scatter plot (A) and violin plot (B) showing the expression of other hub genes for all cell types in GSE118828. (C) The morphological distribution and approximate protein levels of other hub genes. [file Image_3.tif]

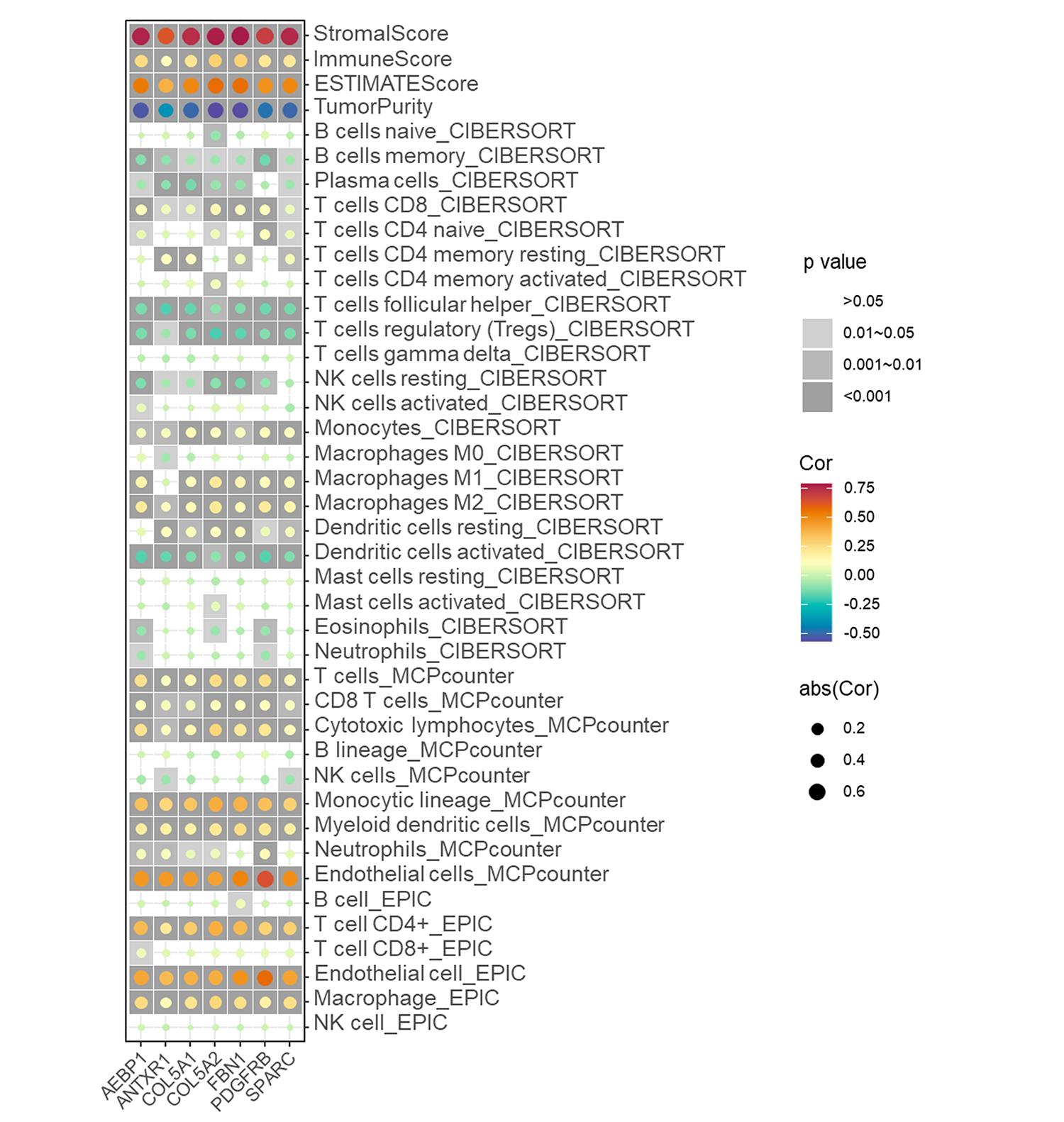

Supplement: Supplementary Figure 4 — The correlations between hub genes and immune cells. [file Image_4.tif]

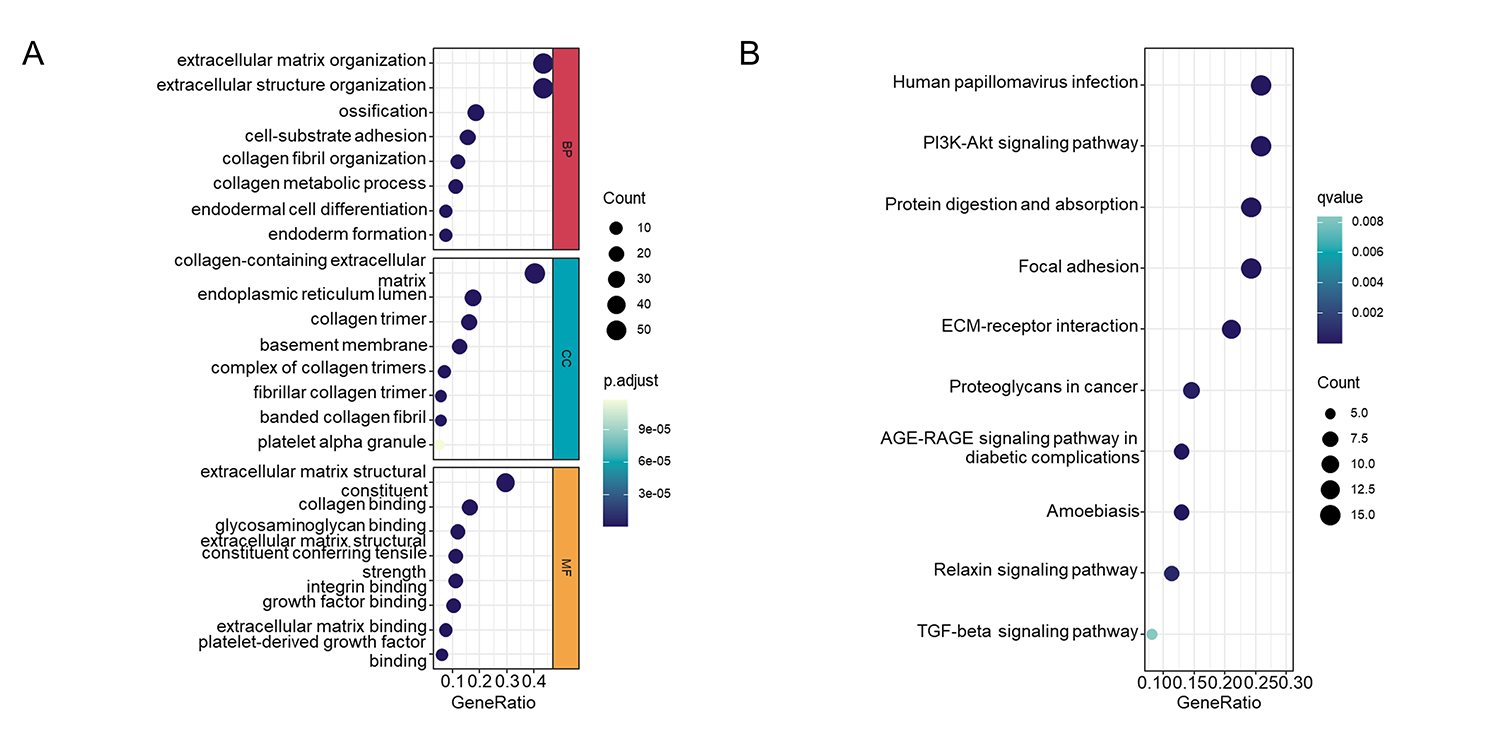

Supplement: Supplementary Figure 5 — Enrichment analyses of CAFs-related genes. (A, B) GO pathways (A) and KEGG pathways (B) in which genes constructing CAFscore were enriched. [file Image_5.tif]

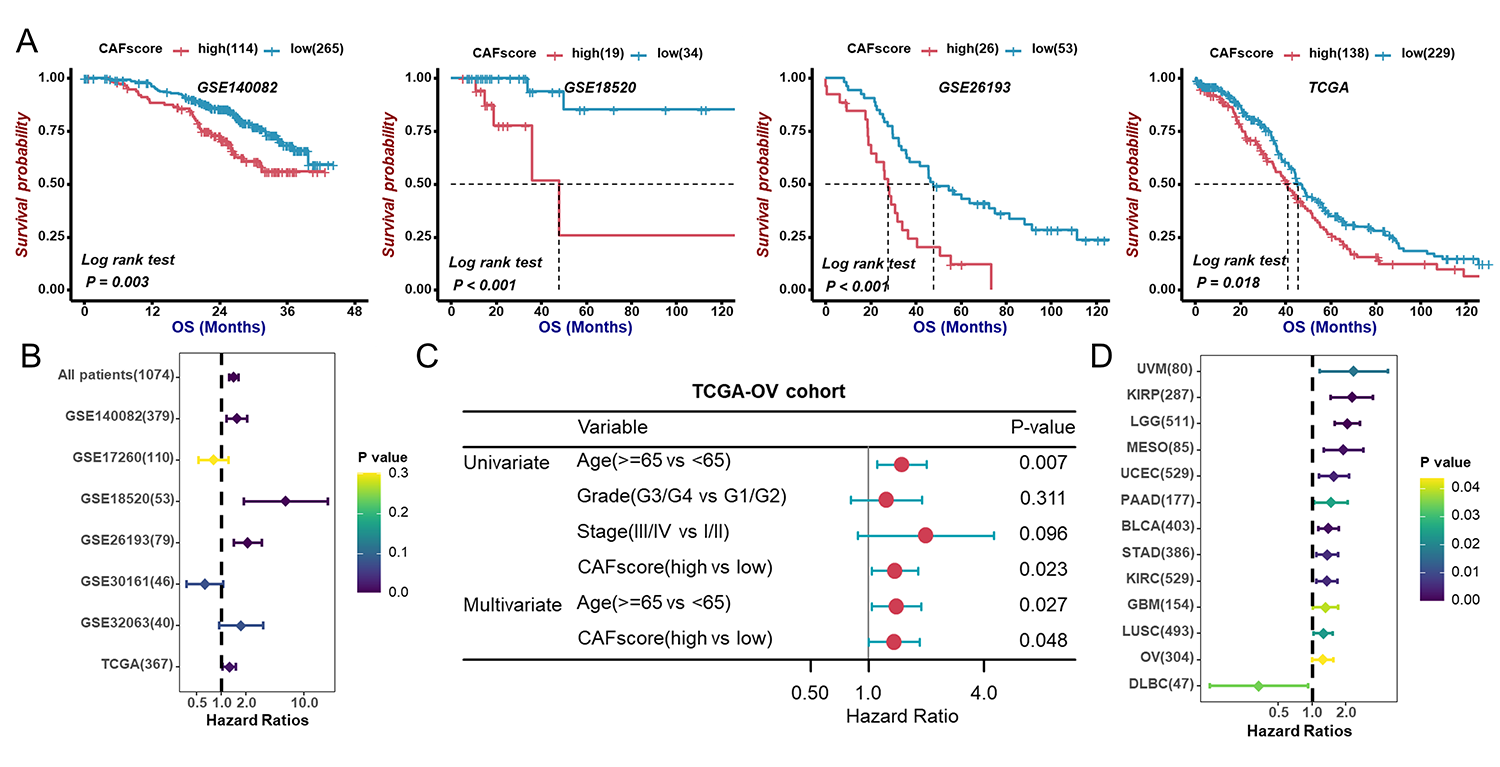

Supplement: Supplementary Figure 6 — The CAFscore is a prognostic indicator in HGSOC. (A) Survival analyses for patient with relatively high- or low-CAFscore in GEO database (GSE140082, GSE18520, GSE26193) and TCGA-OV database using Kaplan–Meier curves. (B) Univariate Cox regression analyses estimating prognostic value of the CAFscore in different HGSOC cohorts. (C) Univariate and multivariate Cox regression analyses of the CAFscore with age, tumor grade and stage in the TCGA-OV cohort. HR and p-values were displayed. (D) Univariate Cox regression analyses estimating prognostic value of the CAFscore in different cancer types from TCGA dataset. [file Image_6.tif]

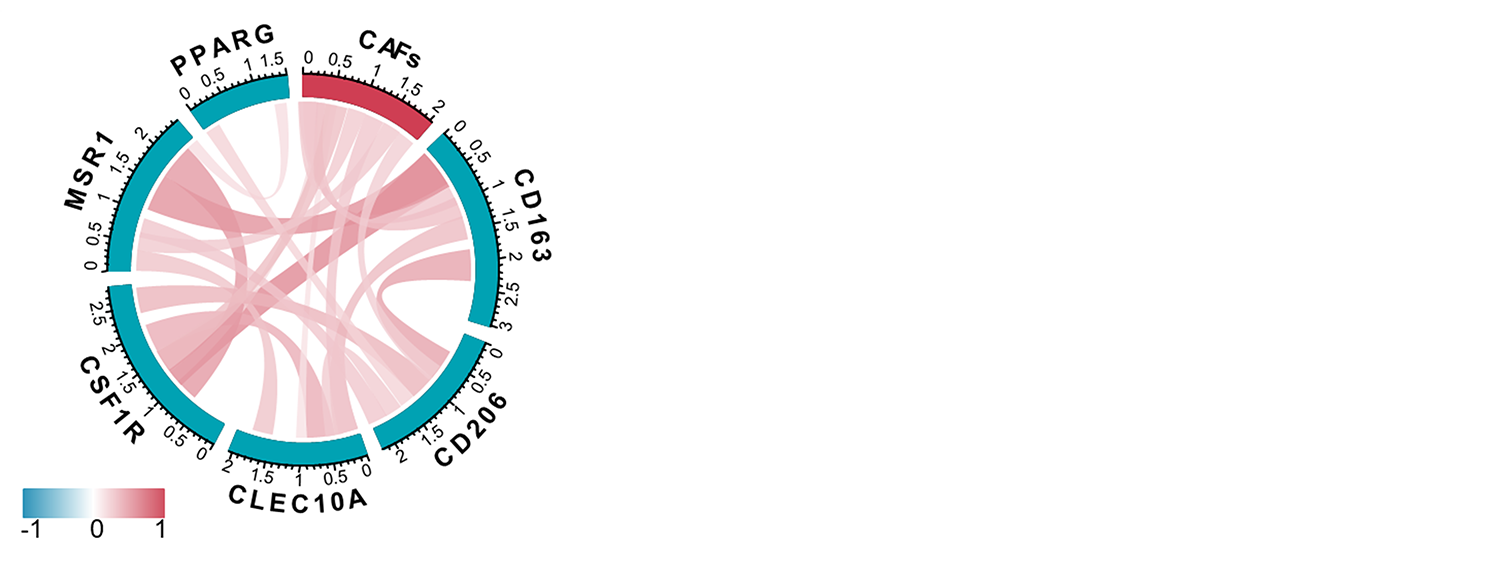

Supplement: Supplementary Figure 7 — The correlations between the CAFscore with the expression of immunosuppressive macrophage markers in mRNA levels. [file Image_7.tif]

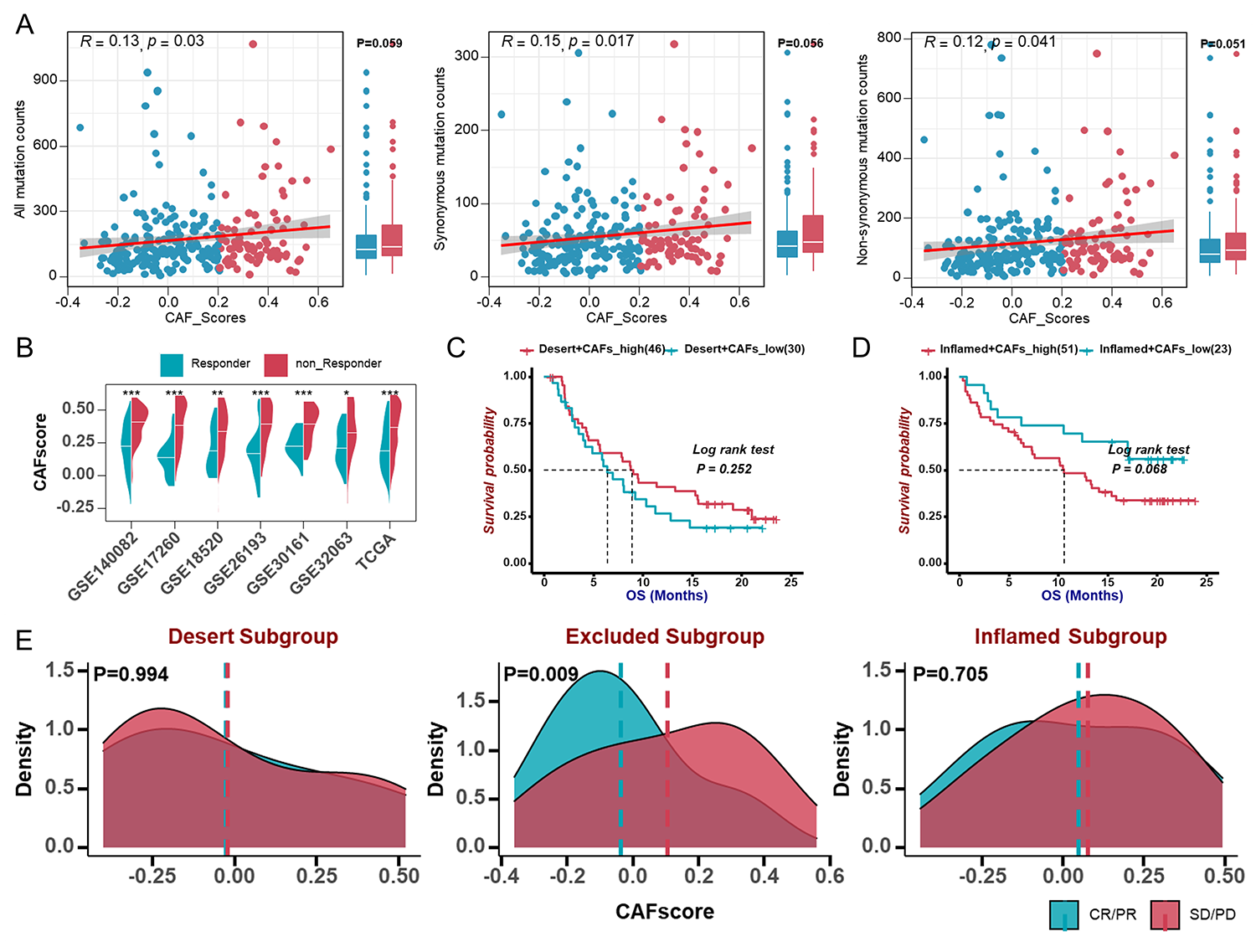

Supplement: Supplementary Figure 8 — The role of the CAFscore in the prediction of immunotherapy benefits (A) The correlations between the CAFscore and synonymous mutation counts, non-synonymous mutation counts, and all mutation counts in TCGA-OV cohort. (B) The comparison of CAFscores between Responder and non-Responder groups in TCGA and GEO datasets, respectively, according to TIDE algorithms. *, P < 0.05; **, P < 0.01; ***, P < 0.001. (C, D) Survival analyses for low and high CAFscore patient groups in the desert (C) and inflamed (D) immune subgroups. (E) The CAFscore in the group with CR/PR versus the group with PD/(SD) in the desert (left), excluded (middle) and inflamed (right) immune subgroups. [file Image_8.tif]
